# The value of dynamic MRI in cervical spondylotic myelopathy: About 24 cases

**DOI:** 10.1016/j.amsu.2022.104717

**Published:** 2022-09-22

**Authors:** Marouane Makhchoune, Michel Triffaux, Triantafyllos Bouras, Sarah Lonneville, Labaisse Marie-Anne

**Affiliations:** a Neurosurgery Department, Hospital Center of Wallonie Picarde, Tournai, Belgium; b Radiology Department, Hospital Center of Wallonie Picarde, Tournai, Belgium

**Keywords:** Cervical myelopathy, Cervical spondylosis, Dynamic, Magnetic resonance imaging, Spinal canal, Case series

## Abstract

Dynamic magnetic resonance imaging (MRI) of the cervical spine is extremely useful in assessing pathological changes at the spinal cord, vertebrae, discs, ligaments and facet joints. We attempted to document the radiological changes that the cervical spine undergoes during dynamic maneuvers and the effects of Dynamic MRI in management of cervical myelopathy, emphasizing on the changes in treatment protocol effected by the new findings discovered. Our work is based on 24 consecutive patients with cervical spondylotic myelopathy had cervical MR imaging in neutral position, in flexion and extension of the cervical spine between January 2021 and December 2021. The result found the mean age was 57.9 years (range 26–85 years). Among these 24 patients, there were 11 males and 13 females. Total number of levels of compression were 47 and the additional levels of involvement were 17. Additional levels of compression were noted in 12 patients, among these 17 new levels, 7 were in the posterior and 10 in the anterior. The most affected level was C5C6 with 16 cases. All additional levels of compression were noted in extension; Reduction of the cervical canal was observed in 20 patients only in extension. In the bending sequences we have noticed an increase of the canal diameter in 3 patients. The location of the compression is in 15 cases anterior, 2 cases posterior and 5 cases are mixed anterior and posterior Surgery was considered in 17 patients. Anterior procedures were 11 (ACDF/corpectomy and fusion) and Posterior surgeries were 6 (laminoplasty/laminectomy), and. The rest of the patients did not require surgery and was conservatively treated. A change of the signal was found in 3 patients during the acquisition in extension position a. Most studies have shown a reduction of the root canal with an increase of the compression level, which was the case in our study. MRI is a useful tool for diagnosis of CM, it does not give an exact idea as to which is the offending level in a multilevel compression that requires surgery. Even the approach and procedure cannot be decided on a static examination and hence are subject to significant interpractitioner the role of extension MRI in determining cervical compression levels. Thus, dynamic cervical spine MRI should be an important investigation before we decide to write off surgical treatment in patients with cervical myelopathy and cord signal changes without definitive compression on static MRI. Flexion and extension MRI is an important tool for decision making and planning appropriate management in cervical compressive myelopathy.

## Highlights

• Dynamic magnetic resonance imaging (MRI) of the cervical spine is extremely useful in assessing pathological changes at the spinal cord. We attempted to document the radiological changes during dynamic maneuvers and the effects of Dynamic MRI in management of cervical myelopathy.
• Most studies have shown a reduction of the root canal with an increase of the compression level, which was the case in our study. MRI is a useful tool for diagnosis of CM, it does not give an exact idea as to which is the offending level in a multilevel compression that requires surgery. Even the approach and procedure cannot be decided on a static examination and hence are subject to significant interpractitioner the role of extension MRI in determining cervical compression levels. Thus, dynamic cervical spine MRI should be an important investigation before we decide to write off surgical treatment.
• Flexion and extension MRI is an important tool for decision making and planning appropriate management in cervical compressive myelopathy.

## 1 Introduction

Degenerative cervical stenosis is considered to be the most frequent disease of the cervical spine during and after middle age. Acquired cervical stenosis is caused by the combination of disc protrusion, facet joints degeneration, hypertrophy of the ligamentum flavum and osteophyte formation These degenerative processes may occur at one or several levels and cause direct cord compromise or secondary ischemic changes by static compression or exacerbated under dynamic movements. Dynamic magnetic resonance imaging (MRI) of the cervical spine is extremely useful in assessing pathological changes at the spinal cord, vertebrae, discs, ligaments and facet joints [1].

Few studies have been done to understand this concept. But all the studies showed that the diameter of the canal is reduced in extension [2]. Dynamic MRI (dMRI) includes separate scans taken with the neck in flexion and extension, and could more accurately identify sites of pathologic stenosis or compression among patients with cervical spondylotic disease [1,2].

Thus we attempted to document the radiological changes that the cervical spine undergoes during dynamic maneuvers and the effects of Dynamic MRI in management of cervical myelopathy, emphasizing on the changes in treatment protocol effected by the new findings discovered.

## 2 Materials and methods

### 2.1 Patients

A 24 consecutive patients with cervical spondylotic myelopathy had cervical MR imaging in neutral position, in flexion and extension of the cervical spine between January 2021 and December 2021. The mean age was 57.9 years (range 26–85 years). Among these 24 patients, there were 11 males and 13 females. Selection of the patients was based on the following inclusion criteria:

• Patients who had clinical signs of myelopathy
• Patients who had cervical canal spondylosis signs on X-ray and MRI
• No other cause of cervical myelopathy was found

### 2.2 Imaging protocol

All patients were examined on a 1.5 T MR unit (Intera, Philips). The SENSE body coil was used. The neutral position MR imaging protocol consisted of sagittal T1-weighted sequence (spin-echo) and sagittal T2-weighted sequence (turbo spin-echo). The cervical flexion and extension angles at which the dynamic MR imaging was done were limited by the cervical spine stiffness of each patient to prevent any neurological deterioration. Each patient chose his maximum comfortable flexion and extension positions, so angles were not the same for all the patients.

## 3 Results

**Fig. 1:**
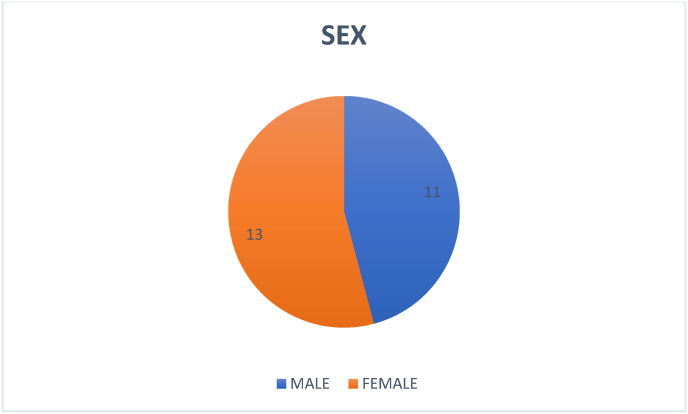
Gender distribution profile.

**Fig. 2:**
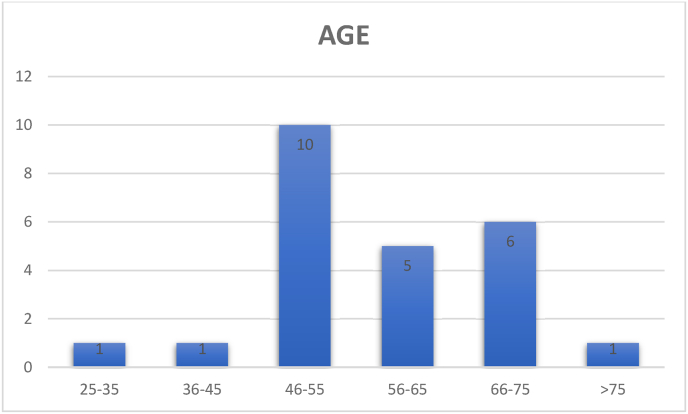
Age distribution profile.

A total of 24 consecutive patients with the diagnosis of CSM presenting to our institution from January to December of 2021 were included in the study. There were 11 men and 13 women and the mean age was 57,9 years (range 26–85 yr) (Fig. 1; 2). Although some patients experienced mild discomfort during the dynamic protocol for MRI acquisition, mainly in cervical extension, none of the patients had any neurological complaints and none of the patients needed to interrupt the examination protocol. There was not excluded patient.

**Fig. 3:**
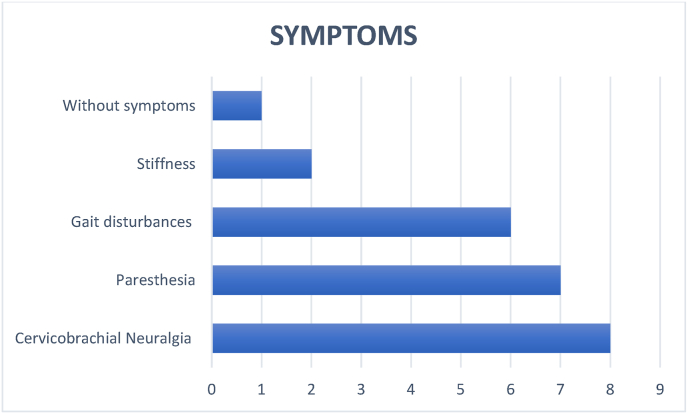
Distribution according to the symptoms.

The symptoms referred were: cervicobrachial neuralgia in 8 cases (33,3%), paresthesia in 7 cases (29,1%), generalized gait disturbances in 6 cases (25%), stiffness in 2 cases (8,3%), without symptoms in one case (4,1%) (Fig. 3).

**Fig. 4:**
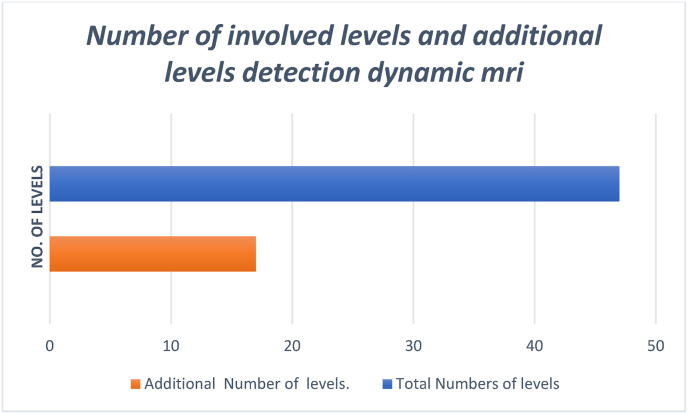
Number of involved levels and additional levels detection dynamic mri.

**Fig. 5:**
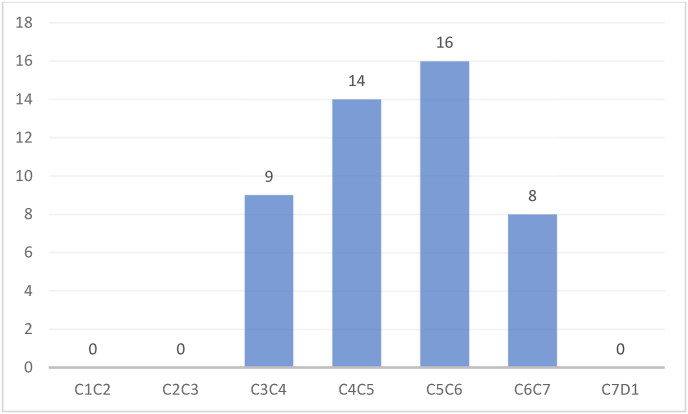
Distribution of stenosis by level.

Total number of levels of compression were 47 and the additional levels of involvement were 17. Additional levels of compression were noted in 12 patients, among these 17 new levels, 7 were in the posterior and 10 in the anterior. The most affected level was C5C6 with 16 cases. All additional levels of compression were noted in extension (Fig. 4; 5).

**Fig. 6:**
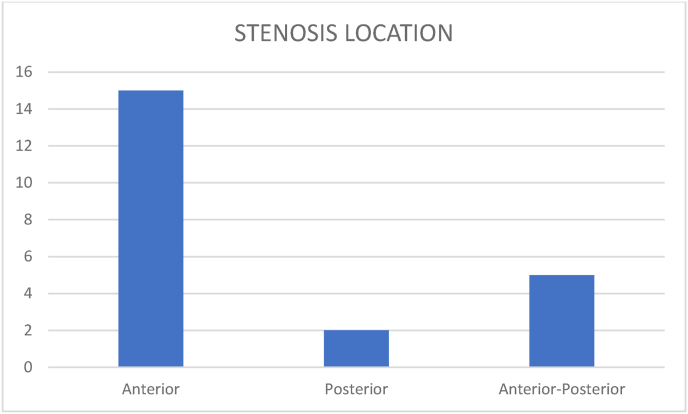
Stenosis location.

Reduction of the cervical canal was observed in 20 patients only in extension. In the bending sequences we have noticed an increase of the canal diameter in 3 patients. The location of the compression is in 15 cases anterior, 2 cases posterior and 5 cases are mixed anterior and posterior (Fig. 6).

Le diamètre moyen en:

- NEUTRE: 7,57 mm
- FLEXION: 8,33 mm soit 10,03%
- EXTENSION: 6,35 mm soit 12,84%

**Fig. 7:**
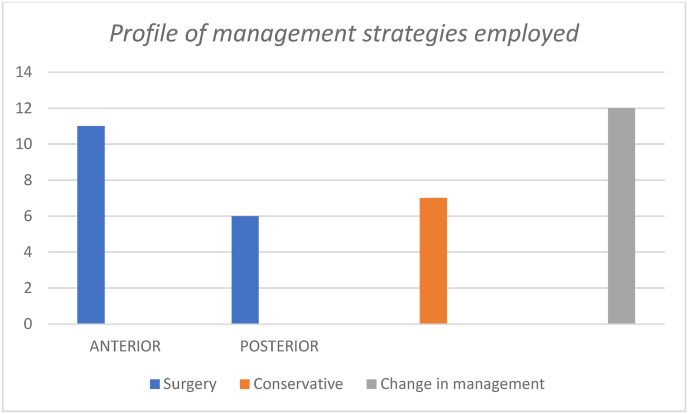
Profile of management strategies employed.

Surgery was considered in 17 patients. Anterior procedures were 11 (ACDF/corpectomy and fusion) and Posterior surgeries were 6 (laminoplasty/laminectomy), and. The rest of the patients did not require surgery and was conservatively treated. However, all the conservatively treated cases were regularly followed up to look for any progression of the symptoms or deterioration. The surgical Management Protocols Were Changed in 12 Patients (Fig. 7).

**Fig. 8:**
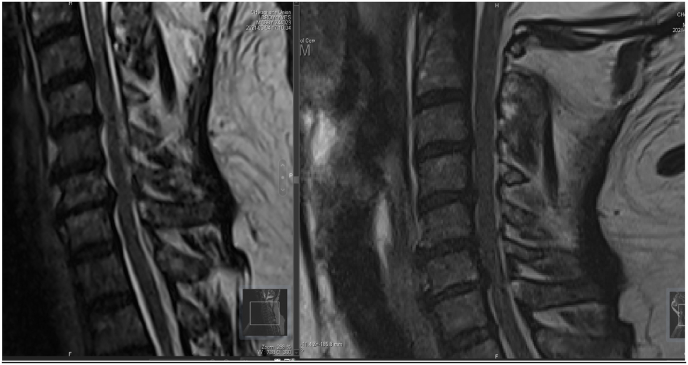
Change of the medullary signal from the neutral position (right) to the extension (left).

**Fig. 9:**
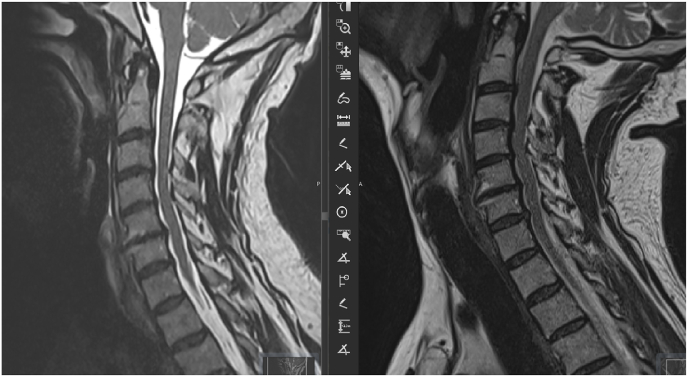
Change of the medullary signal from the neutral position (right) to the extension (left).

**Fig. 10:**
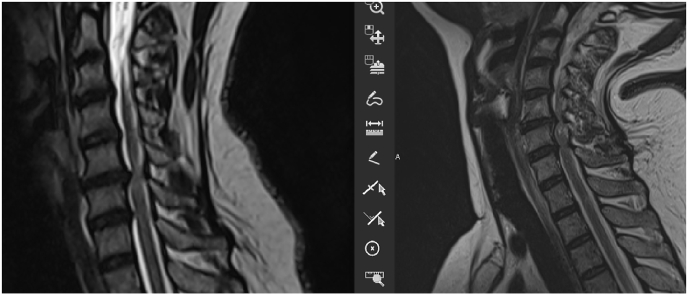
Change of the medullary signal from the neutral position (right) to the extension (left).

A change of the signal was found in 3 patients during the acquisition in extension position (Fig. 8, Fig. 9, Fig. 10).

### 3.1 Case example

**Fig. 11:**
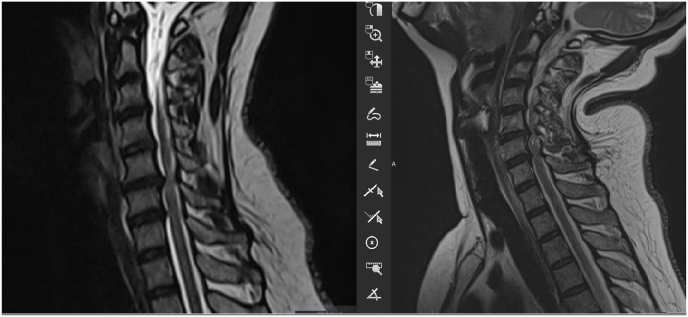
Dynamic MRI shows severe ductal stenosis in C5C6 (right) and the appearance of multiple stenosis a from C3 to C7 in extension (left).

A 49 years old female patient presented with Bilateral upper limb numbness and difficulty in walking. On physical examination, the patient had spastic gait and a positive hoffmann's test bilaterally. A static MRI showed an acute ductal stenosis at C5-6 level. As demonstrated in the (Fig. 11), the extension showed a significant increase in the number of levels involved resulting in a change in procedure employed.

TREATMENT: C3 – C6 LAMINOPLASTIE AND ACDF C6C7, as opposed to a single level procedure.

This case series has been reported in line with the PROCESS Guideline [12].

## 4 Discussion

**Fig. 12:**
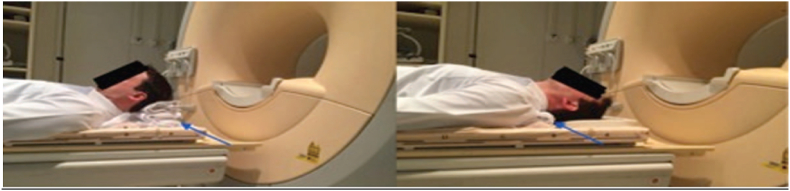
Dynamic MRI.

CSM is a common disease, the pathophysiology of which remains controversial and complex. It has been demonstrated that dynamic factors play an important role in CSM [1]. Flexion and extension result in volume changes in the cervical canal and are likely greater than those in the rest of the spine because of the flexibility of the cervical spine. The dynamic changes are reflected in the spinal cord diameter, transverse cross-sectional area of the spinal cord, and subarachnoid space various authors have performed kinematic MRI studies to define physiologic changes in cervical cord length, variations in the cross-sectional area of the cervical cord, volume of the cervical cord, size of the subarachnoid space, and diameter of the cervical cord Fig. 12 [3]. In our study we have studied the variation of the diameter of the compression on added and its repercussion on the management.

Patients typically examined at 1.5T MRI with special functional positioning device. Despite there are various manufacturers in the current market, most devices are essentially pneumatically driven and mechanically allowing dynamic examination of the spine from the MRI control room [5,6]. All devices allow flexion and extension while a few sophisticated devices allow lateral bending or rotation. The ideal angles used by most studies for flexion and extension were 40–45. However, patients were allowed to stop moving if they experienced pain or neurological deficits [5,6]. The present study provides data on morphometric parameters of cervical spinal canal and cord in patients with CSM based on MRI acquired in the routine neutral position and in flexion and extension positions. The protocol of examination acquisition introduced into our institute is also presented. The maximum degrees of flexion and extension of the neck were based on individual patient tolerance and were predetermined before the examination acquisition, as was the height of the pad to be placed under the head (to maintain flexion of the neck) and under the shoulders (to maintain extension of the neck). Thus, despite mild temporary discomfort reported by a subset of the study patients, all examination acquisitions were successfully completed and there were no neurologic complaints [5].

Muhle et al. [1] used dMRI to evaluate 81 patients aged 30–74 years with different stages of cervical spine degeneration. They observed that patients with complete obliteration of the subarachnoid space and those with severe canal compromise had significant worsening of the canal diameter during flexion/extension. They also observed worsening compression during flexion in patients with multiple disc herniations, caused by a cord draping effect. In patients with very severe stenosis, the motion was quite limited due to facet and ligament hypertrophy. These patients had worsening of the canal diameter in extension [3,6]. It was the case of our study with a canal reduction observe in 20 patients or 83% this reduction is observed in extension.

Raphael R. Pratali found in their study about 18 cases that Spinal canal diameter worsening in extension and increased in flexion, the intraobserver reliability was classified as ‘‘almost perfect agreement’’ (ICC between 0.90 and 0.99, P < 0.001) at each disc space level and in neutral, flexion, and extension positions (Table 1, Table 2). For the SCW, the intraobserver reliability was classified as ‘‘substantial agreement’’ (ICC between 0.79 and 0.96, P < 0.001) at each disc space level and in neutral, flexion, and extension positions.

**Table 1:** Summary of the cervical spinal morphometric parameters measurements, in millimeters.

	Neutral (Mean SD)	Flexion (Mean SD)	Extension (Mean SD)
Longitudinal parameters			
ALSC	108.51 5.90	112.85 6.48	106.19 6.51
PLSC	104.52 5.73	111.11 6.96	100.66 6.56
Transversal parameters			
SCD			
C2–C3	9.9 2.9	10.5 1.9	10.2 2.1
C3–C4	7.7 2.7	8.7 2.6	7.1 2.6
C4–C5	8.0 2.3	9.1 2.3	7.6 2.5
C5–C6	7.8 2.0	8.9 2.0	7.6 2.0
C6–C7	8.4 1.4	9.5 1.5	8.3 1.6

**Table 2:** Intraobserver reliability of spinal canal diameter and spinal cord width.

	SCD			SCW		
	Neutral ICC (95% CI)	Flexion ICC (95% CI)	Extension ICC (95% CI)	Neutral ICC (95% CI)	Flexion ICC (95% CI)	Extension ICC (95% CI)
C2–C3	0.94 (0.86–0.98)	0.95 (0.87–0.98)	0.97 (0.92–0.98)	0.78 (0.41–0.91)	0.91 (0.76–0.96)	0.88 (0.68–0.95)
C3–C4	0.89 (0.73–0.96)	0.98 (0.95–0.99)	0.95 (0.88–0.98)	0.84 (0.58–0.94)	0.85 (0.62–0.94)	0.89 (0.72–0.96)
C4–C5	0.87 (0.66–0.95)	0.94 (0.86–0.98)	0.89 (0.70–0.95)	0.83 (0.55–0.93)	0.89 (0.72–0.96)	0.87 (0.66–0.95)
C5–C6	0.93 (0.83–0.97)	0.93 (0.81–0.97)	0.92 (0.78–0.98)	0.84 (0.57–0.94)	0.94 (0.86–0.98)	0.89 (0.71–0.96)
C6–C7	0.90 (0.75–0.96)	0.83 (0.56–0.93)	0.91 (0.78–0.96)	0.73 (0.29–0.90)	0.82 (0.54–0.93)	0.82 (0.52–0.93)

CI, confidence interval; ICC, intraclass confidence correlation; SCD, spinal canal diameter; SCW, spinal cord width.

Another study done in Istanbul about 258 cases they found On dynamic MR examinations, that the canal widened by an average of 1.05 mm (14.9%) during flexion (8.09 mm) and narrowed by 0.94 mm (13.4%) during extension (6.10 mm) according to the average AP diameter values. The difference between the average flexion and extension MRI results was found to be 1.99 mm. The difference between the MRI measurements and CT measurements was found to be statistically significant (Student's t-test, p < 0.001, Wilcoxon signed-rank test, p < 0.05). Our results were similar with an increase of 10.03% or 8.33 mm in flexion and a reduction of 16.11% in extension or 6.35 mm.

Muhle et al. and Penning and van der Zwaag [1,8]. The cervical subarachnoid space varied from one position to the next. CCAS was largest in the neutral position and smallest in extension. According to Muhle et al. association in extension of shortening of the subarachnoid space, shortening and thickening of the spinal cord, and folding of the ligamenta flava explained the increasing of number of spinal cord impingements that they observed.

Indications of dynamic MR images A previous study showed that dynamic MR scanning was necessary in patients with disc protrusion, osteophyte formation, hypertrophied ligamentum flavum, and a C7 canal. Physicians may prescribe dynamic cervical MR imaging in elderly patients with signs of myelopathy for better evaluation of myelopathy. Our study demonstrates that dynamic MRI found more spinal cord levels compression than static MRI (47 vs. 30) [2].

Although MRI is a useful tool for diagnosis of CM, it does not give an exact idea as to which is the offending level in a multilevel compression that requires surgery. Even the approach and procedure cannot be decided on a static examination and hence are subject to significant interpractitioner variability. Kim et al. [13] investigated the role of extension MRI in determining cervical compression levels. They identified a significantly higher number of compression levels in cervical degenerative myelopathy patients (91%) as compared to the non-myelopathy group (30%). Elderly age was shown to predict increased stenosis in extension. With utilization of dynamic scans, clinician agreement and consensus in interpretation improved [1,3,5,11]. In the present study, we showed that the number of compression levels was increased by 17 levels in extension MR images in 12 or 50% of our patient. This finding indicates that neutral MR images may underestimate the status of cervical spinal stenosis.

A limitation of the present study is the lack of assessment of specific anatomic pathologies that may contribute to spinal cord compression during flexion and extension, including intervertebral disc bulging and ligamentum flavum hypertrophy [5,8,9]. A better understanding of these factors would likely require a higher resolution MRI device (at least 3.0 T). In addition, the present study did not include analysis of spinal cord ischemia from vascular compression or venous congestion, which may occur in different cervical positions during MRI acquisition. Despite the safety and reliability of cervical spine dynamic MRI in patients with CSM, its relevance in treatment choice and planning is not clear and warrants further study [10,12].

24 patients were enrolled in our study had degenerative compressive myelopathy. They had single to multiple level compression. The factors responsible for the compression were apparent in extension of the neck. On subjecting these patients to dynamic MRI study, they showed 17 additional levels in extension views in 12 patients. Most of the symptomatic patients were under the age group of 50–75 yrs. Which is consistent with other data concerning the incidence of cervical myelopathy. Surgery was done in 17 cases of which, of which anterior procedures were considered in 11 cases. Here even a single level ACDF will not only remove anterior compression but also posterior compression by preventing buckling of ligamentum flavum due to increasing the disc height by using grafts. Corpectomy was done in cases where more than one level of anterior compression was present, especially in young patients. Posterior procedures were done in 7 patients where in MRI, the extension showed multilevel involvement with a predominant posterior component. In the elderly, all compression was treated by posterior procedures.

As shown in the survey, extension MR images affected clinical decision-making regarding the number of compression levels, and clinician agreement was improved. We showed that CSM may be better evaluated with extension MR image, especially for patients with signs of myelopathy [3].

## 5 Conclusion

Thus, dynamic cervical spine MRI should be an important investigation before we decide to write off surgical treatment in patients with cervical myelopathy and cord signal changes without definitive compression on static MRI [1,2]. Flexion and extension MRI is an important tool for decision making and planning appropriate management in cervical compressive myelopathy. We recommend it to should be an investigation of choice in all patients of CSM. What changed when we used Dynamic MRI? The extent of the whole disease was apparent: For the first time, hidden levels of compression made apparent on dynamic MRI could be treated appropriately. With the complete extent and site of compression finally visible, the approach to surgery became apparent. Various changes in approach, both surgical and medical, allowed better comprehensive management of the disease ensuring better outcomes and happier patients [2].

## Ethical approval

Written informed consent for publication of their clinical details and/or clinical images was obtained from the patient.

Ethical approval has been exempted by our institution.

## Sources of funding

None.

## Authors contribution

Marouane MAKHCHOUNE: Corresponding author and writing the paper.

Michel TRIFFAUX: writing the paper.

Triantafyllos BOURAS: writing the paper.

Sarah LONNEVILLE: writing the paper.

Marie-Anne LABAISSE: Correcting the paper.

## Trial register number

None.

## Guarantor

MAKHCHOUNE MAROUANE.

## Provenance and peer review

Not commissioned, externally peer reviewed.

## Declaration of competing interest

The authors declare having no conflicts of interest for this article.
